# Vasoplegia: A Review

**DOI:** 10.14797/mdcvj.1245

**Published:** 2023-08-01

**Authors:** Iqbal Ratnani, Rohan Kumar Ochani, Asim Shaikh, Hafsa Nazir Jatoi

**Affiliations:** 1 Methodist DeBakey Heart & Vascular Center, Houston Methodist, Houston, Texas, US; 2 SUNY Upstate Medical University, Syracuse, New York, US; 3 Aga Khan University, Karachi, Pakistan; 4 Dow University of Health Sciences, Karachi, Pakistan

**Keywords:** vasoplegia, vasodilatory shock, septic shock, vasopressors, catecholamines

## Abstract

Vasoplegia is a condition characterized by persistent low systemic vascular resistance despite a normal or high cardiac index, resulting in profound and uncontrolled vasodilation. Vasoplegia may occur due to various conditions, including cardiac failure, sepsis, and post-cardiac surgery. In the cardiac cohort, multiple risk factors for vasoplegia have been identified. Several factors contribute to the pathophysiology of this condition, and various mechanisms have been proposed, including nitric oxide, adenosine, prostanoids, endothelins, the renin-angiotensin-aldosterone system, and hydrogen sulfide. Early identification and prompt management of vasoplegia is crucial to prevent development of shock. This review expands upon the different vasopressors used in management of vasoplegia, including catecholamines such as norepinephrine, dopamine, epinephrine, phenylephrine, and other agents including vasopressin, methylene blue, angiotensin II, hydroxocobalamin, vitamin C, thiamine, and corticosteroids (ie, hydrocortisone). It also emphasizes the importance of conducting further research and making advancements in treatment regimens for vasoplegia.

## Introduction

Vasoplegia, or vasodilatory shock, is a condition defined by profound, uncontrollable vasodilation due to persistently low systemic vascular resistance with normal or high cardiac index.^1^ It can result from a multitude of shock-inducing conditions including, but not limited to, cardiac failure, sepsis, anaphylaxis, hemorrhage, and surgery. However, it most commonly occurs after the development of septic shock^2^ and can manifest as a complication of cardiac surgery in up to 25% of patients.^3^ After the development and recognition of vasoplegia, management must focus on prompt administration of fluids and vasopressors such as catecholamines.^4^

Despite treatment, the development of vasoplegia is an alarming sign of worsening outcomes. It is associated with a significant increase in mortality and morbidity, renal failure, and length of intensive care unit (ICU) and hospital stay.^3,5^ In some cases, catecholamine-resistant vasoplegia also may develop, which has been linked to mortality in almost 25% of patients.^6^ The occurrence of vasoplegia appears to be an independent risk factor for mortality, regardless of the inciting event or procedure, as demonstrated by Tecson et al.’s findings that show twice the risk of mortality in vasoplegic patients after ventricular assist device implantation.^7^ The rate of multiorgan failure and complications, including significant bleeding and respiratory failure, also is associated with increased severity of vasoplegia.^8^ Numerous pathophysiological mechanisms have been proposed as promoting the development of vasoplegia, including endothelial dysfunction due to oxidative injury, elevated levels of endothelin, vasopressin deficiency, overproduction of nitric oxide as well as a foreign body reaction to certain surgical equipment,^9^ making a case for a complex multifactorial pathophysiology. Moreover, it also has been suggested that its respective mechanism of action may be influenced by a preceding condition and might differ, for instance, between septic shock or post-cardiac surgery.^4,9^

Considering the common occurrence of vasoplegia, especially in high-risk patients with preceding fatal conditions at presentation (such as sepsis and the need for cardiopulmonary surgery), one assumes that detailed guidelines outlining management strategies and outcome-improving approaches would have been developed. However, no such evidence-based guidelines exist.^10^ Furthermore, although recent research has shown some initial evidence regarding the benefits of certain treatment strategies, including the use of non-catecholamine vasopressors, methylene blue, angiotensin II, hydroxocobalamin, and ascorbic acid, their efficacy and safety in patients with vasoplegia is still unclear and warrants further investigation.^11,12,13^

In addition to the need for well-studied treatment strategies, risk factor models are needed to predict or evaluate the risk for developing vasoplegia in clinical settings in patients with predisposing conditions. A reasonably sensitive predictive model (area under curve 0.76) was recently developed that could identify patients potentially at risk for vasoplegia based on specific clinical criteria, such as central venous pressure, systolic blood pressure, and the preoperative interagency Registry for Mechanically Assisted Circulatory Support (INTERMACS) profile.^7^ However, it also needs further investigation.

Hence, the case for future research to derive clinical insights, develop outcome-improving guidelines, and construct a well-evaluated standard of care is strong; however, before this can be done in the most conducive manner, it is also essential to understand and consolidate the medical literature. A thorough assessment of the literature is needed—outlining all previous, current, and proposed treatment strategies, the various impacts of these treatment approaches on outcomes, the different pathophysiological mechanisms implicated, and the challenges to current and future research. Considering the current lack of consolidated guidelines, this could be of immense benefit to clinicians searching for rapid answers to clinical conundrums concerning vasoplegia. With that in mind, we conducted this review to qualitatively quantify vasoplegia, its implications, and the most effective strategies developed to date.

## Clinical Risk Factors

Multiple risk factors for vasoplegia have been identified in the cardiac cohort. These include dialysis-dependent renal failure, preoperative medications such as angiotensin converting enzyme (ACE) inhibitors or beta-blockers, combined coronary artery bypass grafting (CABG) and valve procedures, and the need for vasopressors or decline in mean arterial pressure (MAP).^14^ The risk factors for cardiac surgery patients can be divided into pre-, peri- or postoperative parameters. They have been summarized in Table 1.

**Table 1:** Vasoplegic risk factors during cardiopulmonary bypass. BMI: body mass index; LVAD: left ventricular assist device; ACE: angiotensin converting enzyme


Left ventricular ejection fraction > 40%

Male sex

Elderly

Higher BMI

Presence of LVAD

Duration of cardiopulmonary bypass

Immediate hypotension after cardiopulmonary bypass initiation

ACE inhibitor administration perioperatively

Infective endocarditis


### Preoperative Parameters

Dayan et al. report that renal failure was the only significant preoperative risk factor for vasoplegia in patients undergoing cardiac surgery (OR 1.47; 95% CI, 1.17-1.86). The underlying mechanism, although uncertain, can be attributed to elevated proinflammatory cytokine levels due to prevalence of chronic inflammation in this demographic as well as endothelial dysfunction in chronic kidney disease (CKD)—which likely resulted in diminished physiological response to maintain vascular tone—and the consequent induction of NO and cyclic guanosine monophosphate-mediated severe vasodilation.^3,14,15^ Diabetes (OR 1.29; 95% CI, 0.92-1.81) and low left ventricular ejection fraction (mean difference -3.24%; 95% Cl, -9.06% to 2.58%) were also linked to an increase in incidence of vasoplegia preoperatively; however, the association was not statistically significant.^14^

The effects of preoperative use of ACE inhibitors and beta-blockers last for the duration of cardiac surgery and continue their impact postoperatively. ACE inhibitors result in vasoplegia due to an increase in bradykinin and a decrease in the concentration of angiotensin II, which results in reduced vascular tone and blood pressure, and hence vasoplegia.^16,17^ However, research shows a discrepancy regarding the link between the use of ACE inhibitors and the development of vasoplegia. Dayan et al. reported no significant association, while Murat et al. reported a 5-fold increase in the risk of developing vasoplegia following the use of ACE inhibitors.^14,16^ This inconsistency is likely due to the difference in dose and type of ACE inhibitor or differing effects on the renin-angiotensin-aldosterone system.^16^

### Perioperative and Postoperative Parameters

Among the perioperative and postoperative risk factors, combined CABG and valve surgery were most significantly linked to a higher incidence of vasoplegia (OR 2.12; 95% CI, 1.82-2.47); however, aortic graft surgeries were found to be protective against vasoplegia development, likely due to the perioperative hypothermic management (OR 0.48; 95% CI, 0.29-0.78). The core temperatures of 13°C to 16°C used during the surgery provide neuroprotection during circulatory arrest; these extreme temperatures blunt the effect of inflammatory cytokines recruited during cardiopulmonary bypass (CPB).^14,17^

The results from a retrospective study by Leven et al., including 2,823 cardiac patients, suggested that the decline in MAP is highly linked with the development of vasoplegia: 1,645 patients (58.3%) were reported with a considerable decrease in MAP postoperatively (OR 1.26; 95% CI, 1.12-1.43).^17^ This is explained by the possibility of overexpression of inflammatory cytokines on exposure to the CPB circuit, which together with a hypersensitivity reaction triggered by exposure of blood components to foreign surfaces led to an abrupt decline in MAP.^17^

Additionally, the duration of CPB (exceeding 180 minutes), previous history of cardiac surgery, and greater use of red blood cell transfusion are increasingly associated with vasoplegia due to their effect of increasing inflammatory cytokines.^14,15^

### Pathophysiology

Cardiac vasoplegia syndrome has become a prevalent side effect of postcardiac surgery requiring a cardiopulmonary bypass. Substantial hypotension, high or normal cardiac outputs, low systemic vascular resistance, and an elevated need for vasopressors typically characterize this. The complicated interactions between plasma proteins, leukocytes, platelets, and endothelial cells are reflected in this condition. Many enzyme pathways, systemic inflammatory mediators, and neurohumoral factors are triggered by surgical trauma, cardiopulmonary bypass use, and blood exposure to foreign surfaces in the pump and tubing.^18,19^

**Figure 1:**
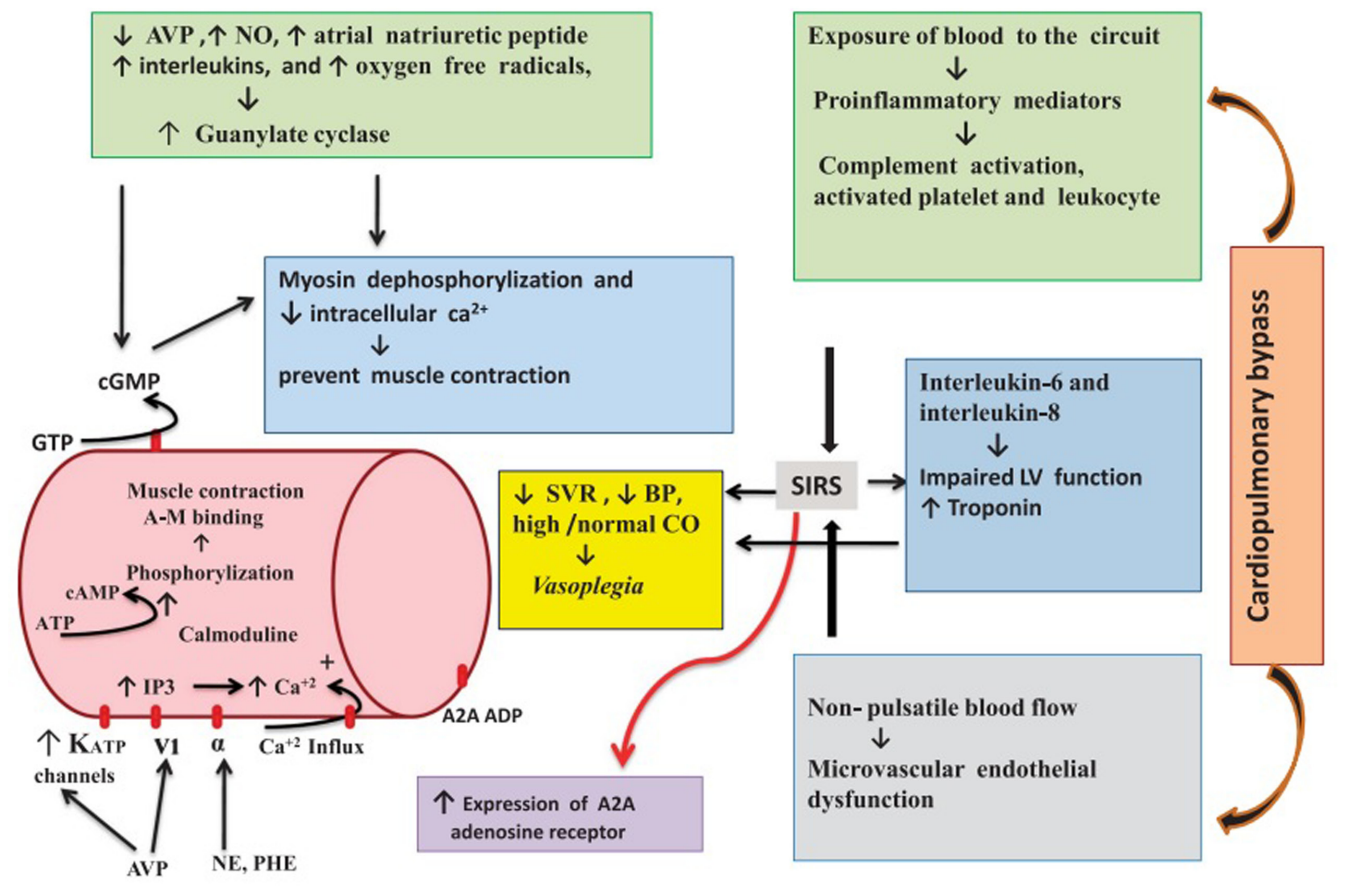
Pathophysiology of postcardiac vasoplegia syndrome. AVP: arginine-vasopressin; NE: norepinephrine; PHE: phenylephrine; V1: vasopressin receptor; α: alpha receptor; A2A ADP: adenosine receptor; NO: nitric oxide; SVR: systemic vascular resistance; CO: cardiac output; BP: blood pressure; LV: left ventricular; SIRS: systemic inflammatory response syndrome; A-M: actin-myosin; IP3: inositol triphosphate; KATP: adenosine triphosphate-sensitive potassium channel; ATP: adenosine triphosphate; cAMP: cyclic adenosine monophosphate; GTP: guanosine triphosphate; cCMP: cyclic guanosine monophosphate. Reprinted with permission from Elsevier.

**Figure 2:**
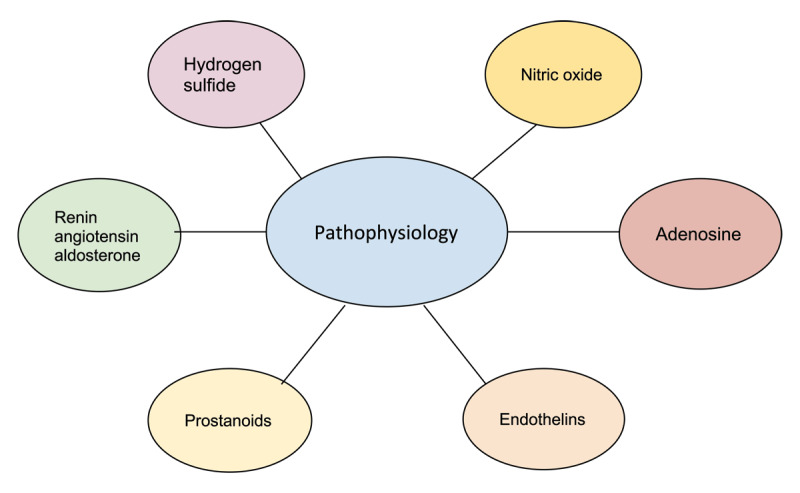
Factors leading to the pathophysiology of vasoplegia.

The pathophysiology of vasoplegia depends on several factors, and studies have shown various mechanisms, including NO, adenosine, prostanoids, endothelins, renin-angiotensin-aldosterone system, and hydrogen sulfide (Figures 1, 2). Various substances such as platelets, macrophages, leukocytes, and neutrophils are also activated during a cardiopulmonary bypass. The neutrophils release proteolytic enzymes activating reactive oxygen species, which in turn attach to membrane/endothelial surfaces. Activated platelets attach to other platelets, neutrophils, and basement membranes, and the activated macrophages secrete cytokines that activate neutrophils and lymphocytes.^4,18,19^

Nitric oxide (NO), initially referred to as the endothelial-derived relaxing factor, diffuses easily to neighboring smooth muscle cells and leads to vasodilation, platelet activation, and leukocyte adhesion. It is formed via L-arginine by endothelial NO synthase. The inflammatory substances, which include bradykinin and thrombin released during an inflammatory process, cause the increased production of NO. NO stimulates vasodilation in a variety of ways,^20,21,22,23^ one of which is activating guanylyl cyclase, found in blood vessel smooth muscle. When NO binds to the enzyme’s heme component, guanylyl cyclase converts guanosine triphosphate to cyclic guanosine monophosphate (cGMP). In turn, cGMP inhibits calcium ion influx through voltage-gated channels and activates cGMP-dependent protein kinases, causing myosin light chains to be dephosphorylated, thereby causing muscle relaxation. Furthermore, NO activates ATP-sensitive potassium channels, resulting in a hyperpolarized state. Despite the activation of G-protein coupled receptors, this state prevents the vasoconstrictive secondary intracellular cascade from occurring.^24^ Furthermore, inflammatory cytokines and pathogen-associated molecular patterns, including lipopolysaccharides, induce the synthesis of inducible NOS isoform. This increases two to three times higher than the baseline and is a major driver of acute vascular dysfunction in the shock mechanism.^21,25,26^

### Adenosine

Adenosine, a substance released by sympathetic and poorly myelinated C fibers, is understood to cause vasodilation and low blood pressure by activating myocardial mechanoreceptors.^27^ This mechanism has been observed during vasovagal syncope. Adenosine also can affect baroreflex activation, which can lead to baroreflex dysfunction when adenosine plasma levels are high. Furthermore a study by Kerbaul et al. compared two groups of patients (vasoplegic and non-vasoplegic) and showed no difference in the patients in the vasoplegic group who were treated with angiotensin-converting enzyme inhibitors before surgery.^28^ There also was no difference in troponin levels between the two groups, indicating that high adenosine plasma levels were not associated with myocardial necrosis.^29,30,31^

### Prostanoids

Prostaglandins, secreted by endothelium, significantly increase during an inflammatory process, inducing vasodilation. This increased secretion of prostacyclins leads to the formation of various other inflammatory stressors, including tumor necrosis alpha, interleukin factor 1, and increased formation of prostaglandin I2 by prostacyclin synthase, eventually leading to vasoplegia.^32,33,34,35^

### Endothelin 1

Endothelin, a predominant isoform of the endothelin family, acts as a vasoconstrictor. It activates the ETA receptors in smooth muscle cells, which leads to an influx of calcium ions and the cells’ contraction. In addition, ETB receptors, also found in smooth muscle cells, are activated as an autoregulatory mechanism to control basal tone via vasodilation and smooth muscle contraction. However, ET1 can potentially lead to harmful effects in extreme inflammatory stress by activating various signaling pathways, including the synthesis of interleukin (IL)-1, IL-6, and tumor necrosis factor.^36,37,38^

### Renin-Angiotensin-Aldosterone System

The renin-angiotensin-aldosterone system (RAAS) can play a role in vasoplegia by affecting vascular tone. RAAS is a hormone system that regulates blood pressure and fluid balance. It is caused by the release of renin from the kidneys in response to low blood pressure or blood volume, which causes angiotensinogen to be converted to angiotensin I. Then, the ACE converts angiotensin I to angiotensin II, a potent vasoconstrictor that can raise blood pressure by constricting blood vessels.

In vasoplegia, the RAAS system may become dysregulated, leading to a decrease in the responsiveness of blood vessels to angiotensin II. This could be due to angiotensin II receptor downregulation, which reduces angiotensin II’s ability to induce vasoconstriction. Furthermore, high levels of NO or other vasodilators may counteract angiotensin II vasoconstriction effects, contributing to the low vascular resistance observed in vasoplegia.^39,40^

### Hydrogen Sulfide

Vasoplegia can be caused by hydrogen sulfide (H_2_S), produced during homocysteine metabolism that requires vitamin B6. Inflammatory states can result in high levels of H_2_S, activating ATP-sensitive potassium (KATP) channels and leading to hyperpolarization, reducing vascular tone. This mechanism is similar to the NO-mediated pathway of vasoplegia, and H_2_S may also synergize with NO in inducing vasodilation.^41^

## Treatment

Prompt management of vasoplegia before the development of shock is crucial and should primarily focus on identification; under ideal conditions, fluid resuscitation with intravenous fluids and cardiac function management must be performed before administering vasopressors.^42^ The vasopressors used under such conditions include catecholamines such as norepinephrine (NE), dopamine, epinephrine, phenylephrine, and other agents, including vasopressin, methylene blue, angiotensin II, hydroxocobalamin, vitamin C, thiamine, and corticosteroids like hydrocortisone. These therapeutic options are depicted in Table 2.^42^

**Table 2:** Therapeutic options for management of vasoplegia after cardiac surgery.^1,10,42^ NO: nitric oxide; V1: vasopressin; AT1: angiotensin 1


TREATMENT OPTION	RECOMMENDED DOSAGE	MECHANISM OF ACTION

**Catecholamines**		

Norepinephrine	0.01-0.2 µg/kg/min	Strong α-1, α-2, and mild β-1 adrenergic agonist

Epinephrine	0.01-0.5 mcg/kg/min	Strong α-1, α-2, and β-1 adrenergic agonist

Dopamine	0-20 mcg/kg/min	α-1 adrenergic agonist at higher doses (dose-dependent agonism)

Phenylephrine	0.5-5 µg/kg/min	α-1 and α-2 adrenergic agonist

**Non-catecholamine agents**		

Vasopressin	0.03 U/min	Acts on *AVPR1a* receptor to reduce vasodilatory effects of NO; V1 agonist

Methylene blue	1.5-2.0 mg/kg/bolus over 15 to 30 minutes	Inhibits guanylyl cyclase and endothelial NO synthase

Angiotensin 2	Start 20 ng/kg/min	AT1 agonist; stimulates release of vasopressin from posterior pituitary gland and release of aldosterone from adrenal gland

Hydroxocobalamin	5 g infusion over 5 min	Inhibits guanylyl cyclase, endothelial NO synthase, and hydrogen sulfide

Ascorbic acid	6 g IV bolus/day	Acts as a cofactor for catecholamine synthesis

Hydrocortisone	50 mg bolus every 6 h or 100 mg every 8 h (10 mg/h)	Hydrocortisone in addition to vitamin C, and thiamine decrease required dose for vasopressors; improves catecholamine response


### Catecholamines

Patients with distributive shock are initially managed with volume expansion and administration of vasopressors like catecholamines with alpha-1-agonist function (epinephrine, NE, dopamine, phenylephrine) to prevent organ dysfunction. These successfully restore blood pressure in most patients, but some patients may show vasopressor resistance.^42^ High doses of vasopressors are required to bring about a significant effect that can yield serious side effects, including peripheral or mesenteric ischemia and increased oxidative stress.^1,4^ In case of vasopressor resistance, another drug with a different mechanism of action must be administered.^4^

Among these catecholamines, NE shows improved (compared to dopamine) or equal (compared to epinephrine and phenylephrine) efficacy and clinical outcomes. Hence, NE is reported to be the first-line agent used in cardiac surgery.^1,4^ It increases MAP and cardiac index without a significant increase in heart rate, thereby reducing myocardial oxygen consumption.^1^ NE is also a β1 adrenergic agonist, unlike phenylephrine, whereby NE maintains ventricular-arterial coupling.^1^ The therapeutic dose of NE for MAP maintenance is 0.2 µg/kg/min, but higher doses of 0.5 to 2 μg/kg/min have reportedly been used as well.^1^

### Non-catecholamine Agents

In case of the inability to maintain blood pressure despite use of high-dose catecholamines, or increased incidence of adverse effects due to catecholamines, other therapeutic agents with a different mechanism of action, such as vasopressin, may be added to the regimen.^1^

Vasopressin is considered a second-line agent for adjunctive therapy in the treatment of cardiac surgery.^1^ A dose of up to 0.03 U/min may be added to raise MAP or to reduce NE dose. Vasopressin acts as an exogenous source of vasoconstrictor, which raises MAP in case of endogenous vasopressin deficiency as in the case of shock. It restores vascular tone to increase blood pressure by binding to the arginine vasopressin receptor 1A gene (*AVPR1a*), *AVPR1b, AVPR2*, oxytocin, and purinergic receptors. Vasopressin acts on *AVPR1*, deactivating the KATP channel directly and inhibiting the increase in cGMP caused by NO. It also reduces the synthesis of NO and decreases the effects of hyperpolarization of the membrane, myosin dephosphorylation, and NO buildup.^10^

Methylene blue is also an important therapeutic agent from among non-catecholamine options. A bolus of 1-2 mg/kg is administered for 10 to 20 minutes or 1 hour as a therapy for distributive shock. Continuous infusion of 1 mg/kg per hour has proven useful following a bolus of up to 48 to 72 hours without a significant decrease in splanchnic circulation.^10^ Methylene blue primarily suppresses vasodilatory effects of cytokines produced in case of shock via inhibition of NO synthase and guanylyl cyclase.^43^ Additionally, methylene blue is a cholinesterase inhibitor via M3 receptor binding.^42^ According to a study by Levin et al., methylene blue is a promising agent for lowering mortality and morbidity in case of vasoplegia following cardiac surgery.^43^

Angiotensin II produced by the liver is responsible for the vasoconstrictive effects of the RAAS via its impact on the AT-1 receptor on vascular smooth muscle.^42^ It also causes an increase in aldosterone release, increases vasopressin release from the posterior pituitary, and increases the body’s sympathetic response.^42^ It is considered a treatment option for vasoplegia in case high-dose vasopressors fail and in case of severe mesenteric ischemia due to high-dose catecholamines since angiotensin II can mobilize venous blood from mesenteric arteries and stimulate clearance of lactate.^10^ A dose of 20 ng/kg is used initially for continuous infusion. In a randomized control trial of 344 people, it was reported that angiotensin is highly effective at increasing blood pressure in patients with distributive shock (adjusted HR 0.44; 95% CI, 0.24-0.80; *P* = .007).^39^

Hydroxocobalamin, a vitamin B12 derivative, is a potent NO synthase and guanylate cyclase inhibitor and may be considered an NO scavenger.^10,42^ Previously used for cyanide and carbon monoxide toxicity, it has shown promising results in vasoplegia following cardiac surgery.^10,42^ In addition to oxygen and vitamin C, hydroxocobalamin can suppress NO activity via hydrogen sulfide production.^10^ A 5 g bolus dose for 10 to 15 minutes/day is recommended to be repeated for up to 48 hours following cardiac surgery.^10^ It is used here as a last resort when all other therapeutic options fail to provide substantial results.

Finally, ascorbic acid, along with hydrocortisone and thiamine, can be used for vasoplegia to reduce the dose requirements of other vasopressors, possibly to reduce the potential of their adverse effects.^10^ Additionally, ascorbic acid is an essential cofactor in catecholamine synthesis for dopamine-beta-hydroxylase and tyrosine hydroxylase; hence, exogenous administration potentiates the synthesis of vasopressors in the adrenal medulla. Following CPB, vitamin C levels decrease significantly, thus decreasing the synthesis of vasopressors. This effect can be mitigated within 24 hours by exogenous administration of a 6 g bolus/day of vitamin C.^10,41^

## Recent Findings: What’s New in the Literature

Two new rescue therapies have demonstrated clinical efficacy in recently published studies, especially for sepsis-related vasoplegia. These include the previously discussed isolated vitamin C therapy or combination therapy with hydrocortisone, vitamin C (ascorbic acid), and thiamine, termed HAT therapy.^44,45^ The reason behind the consideration of vitamin C therapy is its common deficiency in severely ill patients and its implication in causing hypotension.^46^ However, it should be noted that while vitamin C did show reduced vasopressor requirements in one case series, a pilot randomized controlled trial showed no reduction in time to resolution of vasoplegia.^47^

HAT therapy initially showed promise in patients with severe sepsis and concurrent vasoplegia. While HAT therapy could not reduce survival to hospital discharge, it reduced vasopressor requirement durations by almost four times (18.3 hours in the HAT group as opposed to 54.9 hours in the no-HAT group).^48^ However, the largest HAT trial that recently concluded showed no difference in mortality (22% in the HAT group versus 24% in the no-HAT group) and no difference in ventilator and vasopressor-free days.^49^ It should be noted that the trial investigators pointed out a lack of power and other limitations that may have resulted in insignificant results. Additional adequately powered randomized controlled trials are needed before this question can be answered with utmost certainty.

## Conclusion

Vasoplegia may occur due to various conditions such as cardiac failure, sepsis, and post-cardiac surgery, where a low systemic vascular resistance persists despite a normal or high cardiac index, resulting in profound and uncontrolled vasodilation. Multiple risk factors for vasoplegia have been identified in the cardiac cohort, including dialysis-dependent renal failure, preoperative medications such as ACE inhibitors or beta-blockers, combined CABG and valve procedures, and the need for vasopressors or a drop in mean arterial pressure. Studies have demonstrated various pathophysiology mechanisms, including NO, adenosine, prostanoids, endothelins, the renin-angiotensin-aldosterone system, and hydrogen sulfide. The prompt management of vasoplegia is crucial to prevent the development of shock, and it should primarily focus on identification. Ideally, fluid resuscitation with intravenous fluids and managing cardiac function should be performed before administering vasopressors. The vasopressors used in such situations include catecholamines such as NE, dopamine, epinephrine, phenylephrine, and other agents such as vasopressin, methylene blue, angiotensin II, hydroxocobalamin, vitamin C, thiamine, and corticosteroids like hydrocortisone. However, new research and advances are being conducted to further explore the treatment regimens for vasoplegia.

## Key Points

- The development and promotion of vasoplegia involve several pathophysiological mechanisms, including endothelial dysfunction due to oxidative injury, elevated levels of endothelin, vasopressin deficiency, overproduction of nitric oxide (NO), and foreign body reaction to certain surgical equipment.
- Risk factors for vasoplegia in the cardiac cohort include dialysis-dependent renal failure, preoperative use of medications such as angiotensin converting enzyme inhibitors or beta-blockers, combined coronary artery bypass grafting and valve procedures, and the need for vasopressors or a decline in mean arterial pressure.
- Fluid resuscitation and cardiac function management should be prioritized before administering vasopressors in the management of vasoplegia. Vasopressors commonly used in such situations include catecholamines such as norepinephrine, dopamine, epinephrine, and phenylephrine in addition to other agents.
